# Correcting T2* effects in the myocardial perfusion arterial input function avoids overestimation of myocardial blood flow

**DOI:** 10.1186/1532-429X-18-S1-Q14

**Published:** 2016-01-27

**Authors:** Hui Xue, Michael S Hansen, Sonia Nielles-Vallespin, Andrew E Arai, Peter Kellman

**Affiliations:** grid.94365.3d0000000122975165National Heart, Lung, and Blood Institute, National Institutes of Health, Bethesda, MD USA

## Background

Quantitative myocardial perfusion imaging requires correct measurement of arterial Gd concentration as the input function (AIF). An underestimation of AIF Gd concentration will lead to overestimation of myocardial blood flow (MBF). One source of this underestimation is the T2* effect due to high Gd concentration in the AIF measurement. Despite the short echo time (0.65 ms) used in the dual sequence approach specifically designed to avoid T2* loss, we have found significant loss remains at peak concentrations (5-10 mmol/l). To correct this signal loss in the AIF, a conventional dual sequence AIF acquisition [1] is modified to acquire two echoes per phase encode. The T2* effect can be corrected by modeling the exponential decay between two echoes. To validate the influences of T2* on the MBF estimates, pixel-wise MBF maps are computed using the Gadgetron [2] based inline perfusion flow mapping. For both rest and stress perfusion, the MBF values are compared with or without AIF T2* correction.

## Methods

To validate the hypothesis that T2* effect in the perfusion AIF measurement is not negligible, the AIF acquisition was modified to acquire two echoes at different echo times. Parallel imaging (R = 2) was used to maintain a short imaging duration and is applied to reconstruct two images corresponding to two echo times. These two images were combined to correct for T2* effect by utilizing exponential decay and a novel adaptive algorithm to avoid corruption of the low intensity baseline periods. The T2* corrected image were converted into the Gd concentration and input for the MBF estimation. MBF maps with or without T2* AIF correction were computed for N = 8 stress/rest perfusion studies using our standard saturation recovery dual-sequence imaging protocols with FLASH readout: for AIF, echo times 0.65/1.65 ms, flip angle, interleaved parallel acceleration R = 2, acquired matrix 64 × 36; for myocardial perfusion imaging, 14° flip angle, FOV 360 × 270 mm^2^, 8 mm slice thickness, 3 SAX slices, interleaved parallel acceleration R = 3, acquired matrix 192 × 111, ¾ partial Fourier, temporal resolution 53 ms. Pixel-wise MBF maps were computed inline using a two compartment model. All patient studies were approved by local IRB with written consent. Imaging experiments were performed on a 3T clinical MRI system (MAGNETOM Skyra, Siemens). The administered Gd dose (Gadavist) was 0.075 mmol/kg (FLASH) with an infusion rate of 2 ml/s.

## Results

**Figure 1:**
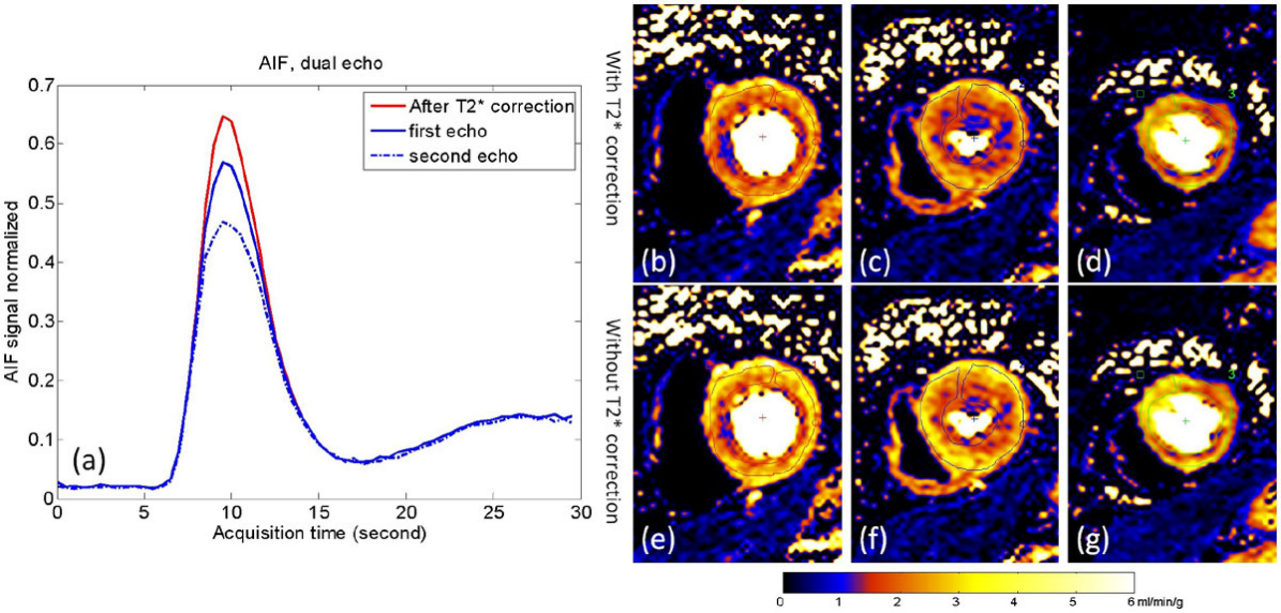
**A stress FLASH perfusion study**. The AIF time intensity curves are shown in (a) for the first and second echo. The curve after T2* correction is also plotted, highlighting the underestimation of AIF if T2* effects are not corrected. The pixel-wise MBF maps are shown in (b-d) for T2) corrected AIF and (e-g) for the uncorrected AIF curve. The underestimated AIF Gd concentration leads to overestimated MBFs. For the three ROIs, the MBF values (ml/min/g) with and without T2* correction are 2.22 ± 0.49, 2.01 ± 0.56, 2.03 ± 5.6, 2.34 ± 0.66.

**Figure 2:**
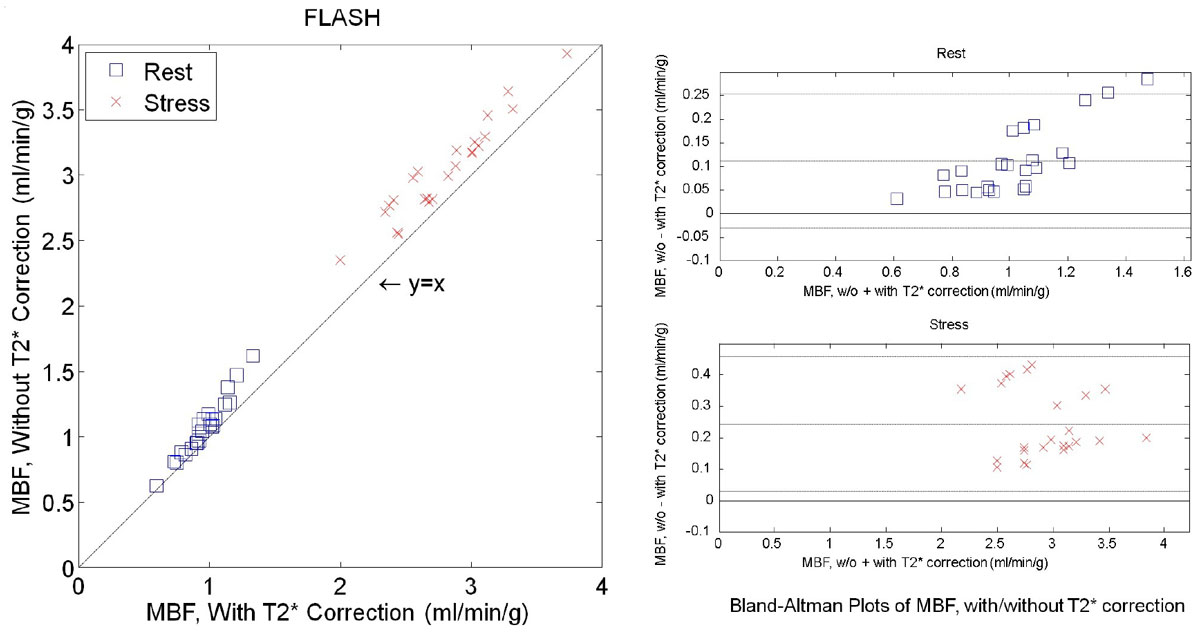
Comparison of MBF estimates with and without T2* correction on AIF. Pixel-wise MBF maps were computed using a two compartment model deconvolution. The mean MBF values for all FLASH rest/stress cases are 0.96 ± 0.16/2.80 ± 0.39 with T2* correction and 1.07 ± 0.22/3.04 ± 0.37 without T2* correction. Significant differences were found for the FLASH studies (t-test, p-value, rest/stress: 0.053/0.030).

An example of rest/stress perfusion studies (Figure 1) plots the AIF signal before and after T2* correction. The corresponding flow maps illustrate the overestimation of MBF if T2* effect is not corrected. Mean MBF overestimates (Figure 2) caused by uncorrected AIF are 9.1 ± 4.9% and 11.1 ± 5.8% for stress and rest, respectively.

## Conclusions

We validated the hypothesis that T2* effects in the perfusion AIF measurement can lead to significant overestimation of quantitative myocardial flow estimates.
